# Appropriate particle size of rice straw promoted rumen fermentation and regulated bacterial microbiota in a rumen simulation technique system

**DOI:** 10.3389/fvets.2023.1185191

**Published:** 2023-06-12

**Authors:** Zhiqing Li, Huijing Qiu, Xinyi Lan, Zuo Wang, Weijun Shen, Fachun Wan, Dingfu Xiao, Jianhua He

**Affiliations:** College of Animal Science and Technology, Hunan Agricultural University, Changsha, China

**Keywords:** rice straw, goat, rumen simulation technique (RUSITEC), rumen microorganisms, rumen fermentation

## Abstract

The purpose of this study is to reveal the effects of different particle sizes of rice straw on the rumen protozoa count, nutrient disappearance rate, rumen fermentation, and microbial community in a rumen simulation technique (RUSITEC) system. In this experiment, a single-factor random trial design was adopted. According to the different particle sizes of rice straw, there were three treatments with three replies in each treatment. Three kinds of goat total mixed ration (TMR), with the same nutrients were used to carry out a 10 days *in vitro* fermentation experiment using the rumen simulation system developed by Hunan Agricultural University, including 6 days the pretrial period and 4 days formal period. This study found that the organic matter disappearance rate, concentrations of total volatile fatty acids (VFAs), acetate, propionate, and iso-butyrate were greatest in the 4 mm group (*p* < 0.05). There were no significant differences in the alpha diversity, among the three groups (*p* > 0.05). The relative abundance of *Treponema* and *Ruminococcus* of the 2 mm group increased; the relative abundance of *Butyrivibrio* and *Prevotella* in samples increased in the 4 mm group. In addition, the results of correlation analysis showed that *Prevotella* and *Ruminococcus* was positively correlated with butyrate, ammonia-N, dOM and d ADF (*p* < 0.05) and negatively correlated with valerate (*p* < 0.05); *Oscillospira* was positively correlated with valerate (*p* < 0.01) and negatively correlated with propionate, butyrate, ammonia-N, dOM and dADF (*p* < 0.05). The present results imply that compared to the other groups, rice straw particle size of 4 mm may improve the disappearance rate of nutrients and promote the production of volatile fatty acids by regulating ruminal microorganisms.

## 1. Introduction

Rice is the world's third most important particle crop following wheat and corn (1). Rice straw is a crucial residue that is generated in large amounts in Asia. The major proportion of the rice straw is directly burnt in the field, which causes air pollution. However, rice straw can be a crucial source of feed for ruminants. According to statistics, China has 133 million goats and 173 million sheep in 2020 (2). Rational utilization of rice straw resources is of great significance for the development of animal husbandry in China.

The rumen simulation technique (RUSITEC) is an *in vitro* fermentation system that simulates the physiological functions of the rumen, which can reduce the limitations of animal experiments *in vivo*. It plays an essential role in studying rumen microorganisms and rumen fermentation mechanisms (3). The RUSITEC used in this study adopts the continuous culture method. Specifically, we can continuously inject buffer solution into the fermentation tank while continuously discharging fermentation products from the fermentation tank to form a continuous and dynamic fermentation system. The standardization of RUSITEC is crucial for the accuracy and comparability of research results on ruminant nutrition.

Currently, there are many studies on technical indicators such as dilution ratio and feed input ratio on RUSITEC (4, 5), while there are few reports on comparative studies of coarse feed particle size. A few *in vitro* studies have shown that feed particle size can affect the fermentation characteristics of RUSITEC. Smaller feed particle sizes, concentrations of acetic acid, total volatile fatty acids, ammonia nitrogen, and dry matter and crude protein loss rates are lower, but total nitrogen content is higher (6–8). In previous studies, the particle size of the feed used by researchers was highly variable, such as 0.45 (9), 1 (10, 11), 3 (12), 4 (13), and 5 mm (14, 15). The feed particle size differed by nearly 10 fold and the comparability of the test results was poor. Therefore, this experiment used RUSITEC to study the effects of three diets (1, 2, and 4 mm) with relatively similar particle sizes of rice straw on fermentation characteristics and rumen microflora in goats, with a view to providing references for the rational utilization of rice straw resources and the improvement of artificial rumen technical indicators.

## 2. Materials and methods

### 2.1. Experimental design and diets

Rice straw was crushed by hammer mill with sieve aperture of 1 mm, 2 mm, and 4 mm, and the crushed rice straw was prepared into three TMR in a 50:50 ratio of concentrate to roughage. The samples were then separately weighed (20 g), stored in a properly sealed plastic bag, refrigerated, and used as the base ration of RUSITEC. The experimental treatments were group 1 mm, group 2 mm, and group 4 mm. The experimental diet and rumen fluid donor goat diet were formulated according to the nutritional requirements of goats in NRC (16).

The RUSITEC consisted of nine fermentation vessels, which were allocated to three groups (three vessels per group); each fermentation vessel was randomly assigned to receive each diet once. Each 10 days period consisted of a 6 days diet adaptation period followed by a 4 days sampling period. The composition and nutritional composition of the diets were shown in Table 1.

**Table 1:** Composition and nutrients levels of the basal diets (DM basis).

**Ingredients**	**Content, %**
Rice straw	50.00
Corn	29.20
Soybean	8.00
Wheat bran	11.09
CaCO_3_	0.11
CaH_2_PO_4_	0.40
NaCl	0.70
Premix^a^	0.50
Total	100
**Nutrient levels** ^b^	
DM	98.70
CP	10.28
NDF	73.99
ADF	45.89
EE	15.06

a One kg of premix contained the following: Vitamin A 15 544 IU, Vitamin D 23 220 IU, Vitamin E 297 IU, Fe 31 mg, Cu 25 mg, Zn 60 mg, Mn 75 mg, I 0.15 mg, Se 0.05 mg, Co 0.15 mg.

b The nutrient levels were all measured values.

CP, crude protein; NDF, neutral detergent fiber; ADF, acid detergent fiber; EE, ether extract; DM, dry matter.

### 2.2. RUSITEC fermentation

Three rumen-fistulated Xiangdong black goats (30 ± 2.5 kg) were used in the experiment. Before morning feeding, contents were collected from the rumen of three goats, mixed and filtered with three layers of gauze. Then the filtered rumen fluid was stored in an insulated container filled with CO_2_. Buffered rumen fluid was prepared by mixing strained rumen fluid with prewarmed McDougall's (17) buffer at a ratio of 1:1 under a stream of CO_2_. Then, 20 g of TMR was added to each fermentation vessel and 1,000 ml of rumen fluid mixed with buffer under anaerobic conditions. Fermentation vessel contents were continuously stirred by a central propeller apparatus driven by magnets at the rate of 25 r/min and the temperature of the fermentation vessel was maintained at 39°C through a circulating hot water bath inside the water jacket in the RUSITEC system.

### 2.3. Sample collection and analysis

#### 2.3.1. Rumen fermentation characteristics

After the start of the test, each day before feeding (8:00 am and 8:00 pm) the contents of fermenters was collected for protozoan counting. First, 5 ml fermentation broth was collected from the sampling port of the fermenter and placed in a 15 ml centrifuge tube. Next, 10 ml of methyl green staining liquor was added and shaken to rest overnight. Then the Protozoan count was performed by Sedgewick-Rafter counting plate and biological microscope.

From the sampling period (day 7), contents of fermenters filtered with three layers of gauze was collected, divided into two tubes with 5 ml frozen storage tube, and stored in a −20°C freezer. Samples were thawed and centrifuged at 15,000 g for 10 min at 4°C, and individual VFAs concentrations were determined by gas chromatography (Agilent 7890A; Agilent Inc.), Wang et al. (18) describe the method. In addition, ammonia in the supernatant was determined colorimetrically according to the method of Wang et al. (19) and Weatherburn (20).

#### 2.3.2. Determination of nutrients disappearance

Before the samples were collected, nitrogen was introduced into the RUSITEC system, the solids and liquids in the fermentation vessel were mixed evenly. Then all the solids and liquids in the vessel were discharged into a 1,000 ml measuring cylinder, the total volume was recorded and the discharged mixture was filtered with a nylon bag, which was cut from nylon cloth with an aperture of 50 μm and a size of 12 cm × 8 cm. The solid effluent of each fermenter within 24 h was collected in the same nylon bag, cleaned, dried, crushed, and stored for determination of routine nutrient content. The contents of dry matter, crude protein, neutral detergent fiber, and acid detergent fiber in feeds and residues were determined according to Van Soest et al. (21).

#### 2.3.3. Microbiota analysis by 16S RNA

Collected the contents of fermenters was collected and put them in the EP tubes after high-temperature sterilization, and then extract total DNA from the contents of fermenters samples using the Qia amp fast DNA Kit (Qiagen, Germany). The DNA extraction was checked on 1% agarose gel, and DNA concentration and purity were determined with Nano Drop 2000 UV-vis spectrophotometer (Thermo Scientific, Wilmington, USA). The hypervariable region V3–V4 of the bacterial16S rRNA gene was amplified by ABI GeneAmp^®^9700 PCR thermo-nuclear (ABI, CA, USA). The PCR product was removed from 2% agarose gel and purified using the AxyPrep DNA Gel Extraction Kit (Axygen Biosciences, Union City, CA, USA) according to manufacturer's instructions and quantified using Quantus ™Fluorometer (Promega, USA). Paired-end sequencing was performed on the Illumina miseq pe300 platform/novaseq PE 250 platforms (Illumina, San Diego, USA). According to previous studies (22, 23), the original 16S rRNA gene sequencing reads were demultiplexed, quality filtered, and merged. Operational taxonomic units (OTUs) with a 97% similarity cutoff were clustered using UPARSE Version 7.1 (24), and chimeric sequences were identified and removed. ACE and Chao richness estimators, Shannon and Simpson diversity indices were used to assess species diversity complexity (25). Beta diversity was assessed using Principal Component Analysis (PCA). An analysis of similarities (ANOSIM) was used to assess significant differences between samples.

### 2.4. Statistical analysis

SAS (Version 9.4, USA) software was used for one-way ANOVA. When the difference was significant, Duncan's method was used for multiple comparisons. Using Spearman correlation analysis, the relationship between the bacterial abundance and the disappearance of nutrients, ruminal ammonia-N and VFAs concentrations was examined. The results were presented as the mean and standard error of means (SEM). Statistical difference was respectively declared as significant or highly significant at *p* < 0.05 or *p* < 0.01.

## 3. Results

### 3.1. Rumen fermentation characteristics

**Figure 1:**
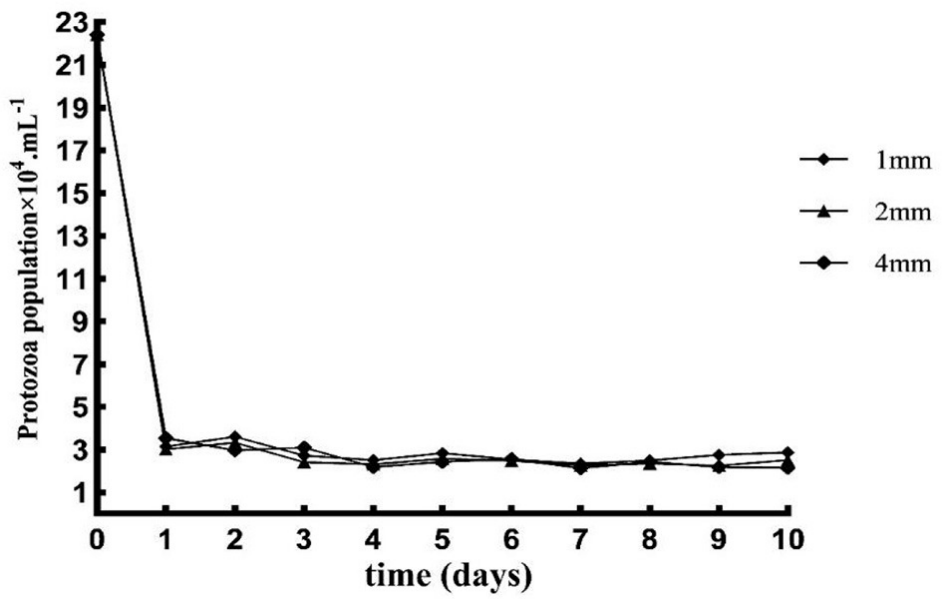
Effects of different straw particle size on the number of protozoan.

The number of protozoa decreased rapidly from day 0 to day 1, slowly from day 2 to day 4, and tended to be stable from day 6 to day 10 (Figure 1).

The concentration of total VFAs, acetate, propionate, and iso-butyrate were higher in the 4 mm group than others (*p* < 0.05; Table 2). In addition, the valerate and A/P ratio of the 4 mm group were significantly lower than other groups (*p* < 0.01).

**Table 2:** Effects of different straw particle size on products of rumen fermentation.

**Units**	**Particle size**			**SEM**	***p*-value**
	**1 mm**	**2 mm**	**4 mm**		
Total VFAs, mml/L	93.03^b^	94.01^b^	97.24^a^	1.27	0.03
**Individual, % of total VFAs**					
Acetate	72.52^b^	72.30^b^	74.42^a^	1.09	<0.01
Propionate	13.05^b^	13.71^b^	14.41^a^	0.13	<0.01
Iso-butyrate	0.76^b^	0.71^b^	0.84^a^	0.02	<0.01
Butyrate	7.79	7.85	7.15	0.35	0.06
Iso-valerate	3.15	2.90	1.92	0.18	0.14
Valerate	2.73^a^	2.53^b^	1.26^c^	0.03	<0.01
A: P	5.56	5.27	5.16	0.16	0.11
Ammonia-N (mg/dL)	5.85	6.14	7.16	0.34	0.07

SEM, standard error of the mean.

^a−*c*^Means within a row for doses that do not have a common superscript differ (p <0.05). Total VFAs = acetate + propionate + butyrate + valerate + iso-butyrate +iso-valerate.

A: P, acetate: propionate.

### 3.2. Determination of nutrients disappearance

**Figure 2:**
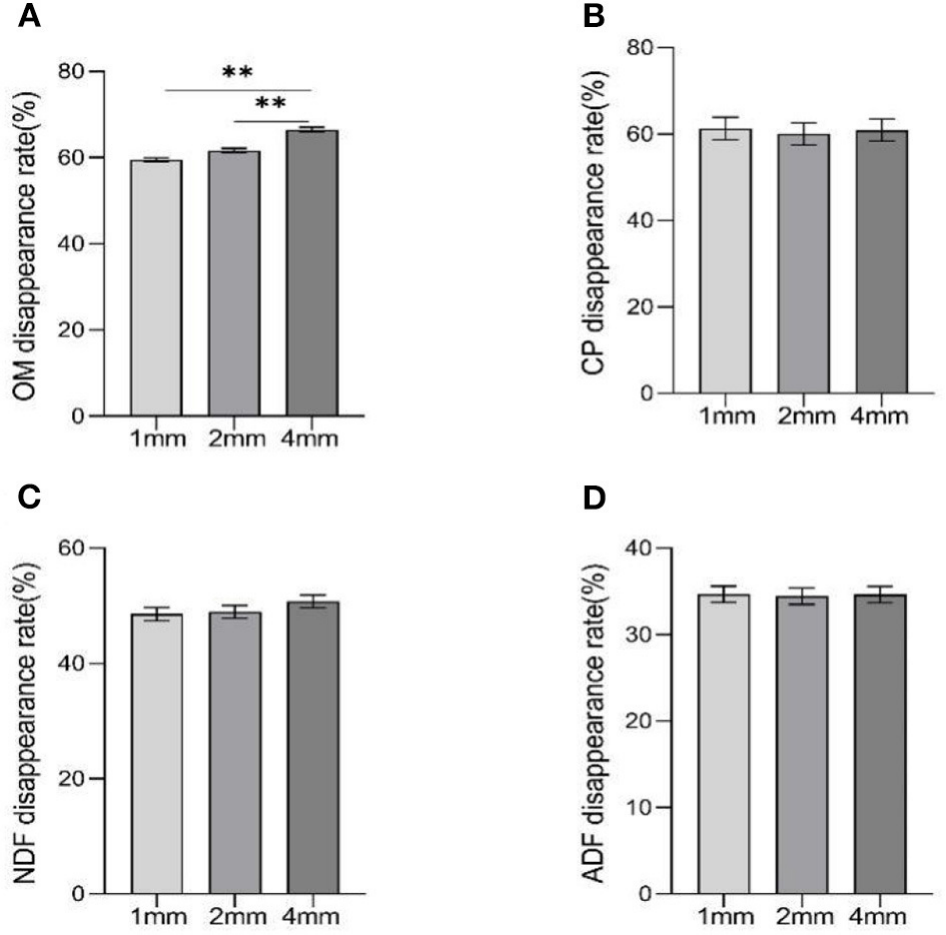
The effects of different straw particle size on disappearance rate of nutrients. Data were shown as means ± SEM, significant (*p* < 0.05) or highly significant (*p* < 0.01) statistical differences were represented by * or **. **(A–D)** are the disappearance rates of OM, CP, NDF, and ADF, respectively.

The disappearance rate of OM was higher in the 4 mm group than other groups (Figure 2, *p* < 0.01), but there was no difference between the 1 mm and 2 mm groups (*p* > 0.05).

### 3.3. Rumen microbial diversity

**Figure 3:**
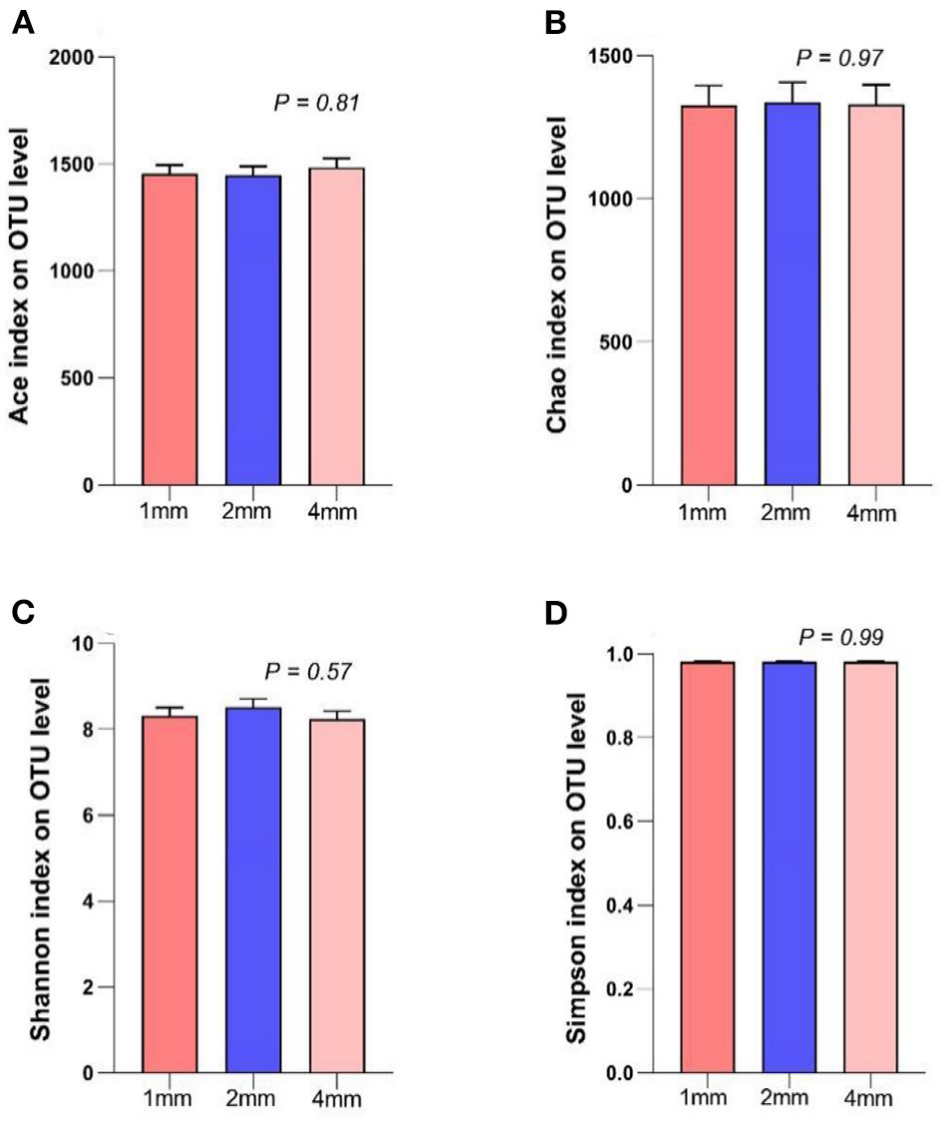
Effects of rice straw particle size on the ruminal microbiota alpha diversity in a rumen simulation technique system. **(A)** Ace index, **(B)** Chao index, **(C)** Shannon index, **(D)** Simpson index.

There were no significant differences in the Ace index, Chao index, Shannon index, Simpson index, among the three groups (Figure 3, *p* > 0.05).

**Figure 4:**
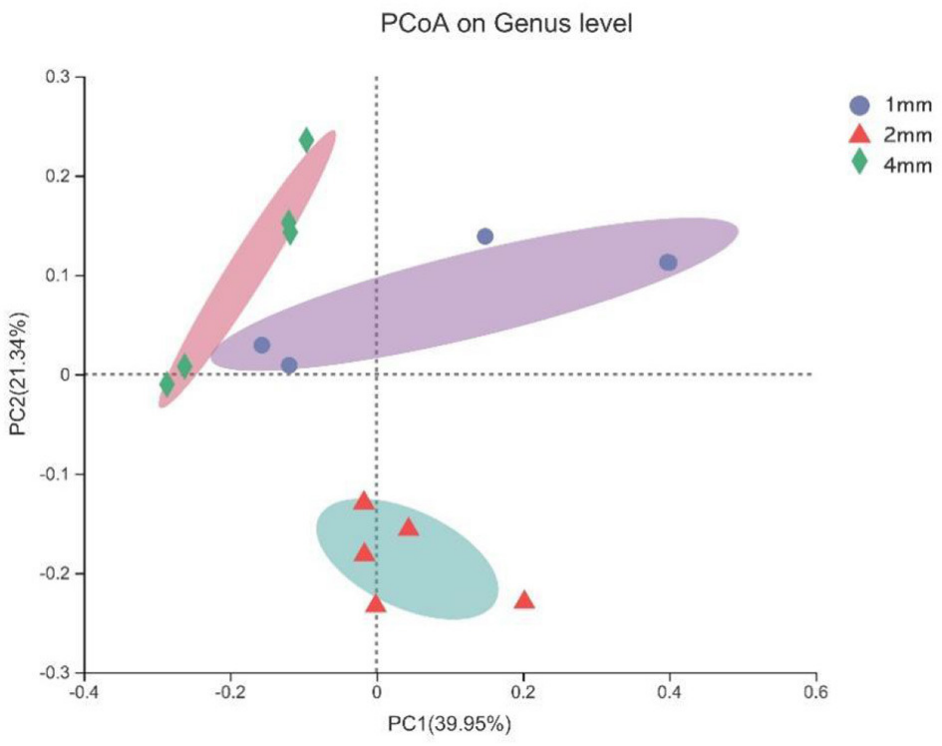
Principle Coordinate Analysis (PCoA) based on Bray-Curtis distance was used to compare and analyze the rumen microbial community composition of three straw particle sizes. The *X*-axis and *Y*-axis correspond to principal components 1 (PC1) and 2 (PC2), which explain the highest level of variation.

The microbial composition of contents of fermenters samples were significantly different in different treatment groups, and the explanation degree of PC1 axis and PC2 axis was 39.95 and 21.34%, respectively (Figure 4).

### 3.4. Relative abundance of rumen microorganisms

At the phyla level, Bacteroidetes and Firmicutes are the main dominant bacteria. The relative abundance of Bacteroidetes was higher in the 2 and 4 mm groups than that in the 1 mm group (*p* < 0.05; Table 3). However, the relative abundance of Proteobacteria, Tenericutes, Cyanobacteria, TM7, and WPS-2 were significantly higher in the 1 mm group than others (*p* < 0.01).

**Table 3:** Effects of different straw particle sizes on rumen bacterial community at phylum level (%).

**Units**	**Particle size**			**SEM**	***p*-value**
	**1 mm**	**2 mm**	**4 mm**		
Bacteroidetes	59.87^b^	63.48^a^	64.96^a^	1.13	0.02
Firmicutes	30.91	30.59	29.13	1.24	0.94
Proteobacteria	1.44^a^	0.81^c^	1.07^b^	0.08	<0.01
Spirochaetae	3.51	2.27	2.09	0.41	0.06
Tenericutes	0.60^a^	0.47^ab^	0.38^b^	0.05	0.03
Cyanobacteria	0.25^a^	0.16^b^	0.14^b^	0.02	<0.01
TM7	0.41^a^	0.29^b^	0.31^b^	0.02	<0.01
WPS-2	0.25^a^	0.15^b^	0.32^a^	0.03	<0.01
Others	2.76	1.78	1.6	0.14	0.07

SEM, standard error of the mean.

^a−*c*^Means within a row for doses that do not have a common superscript differ (p <0.05).

At the genus level, the relative abundance of *Prevotella, Ruminococcus*, and *Butyrivibrio* were the main dominant bacteria, *Prevotella* in the 2 and 4 mm groups were significantly higher than that in the 1 mm group (*p* < 0.01; Table 4). Compared with 1 and 4 mm groups, the relative abundance of Treponema in the 2 mm group increased significantly. Interestingly, the relative abundance of *Oscillospira* and *Mogibacteriaceae* decreased with the increase of straw particle sizes (*p* < 0.01).

**Table 4:** Effects of different straw particle sizes on rumen bacterial community at genus level (%).

**Units**	**Particle size**			**SEM**	***p*-value**
	**1 mm**	**2 mm**	**4 mm**		
*Prevotella*	30.72^b^	40.43^a^	39.61^a^	1.85	<0.01
*Ruminococcus*	14.52	15.01	13.69	4.02	0.12
*Butyrivibrio*	9.20	8.77	5.00	3.89	0.17
*Clostridiales*	4.56	5.05	5.00	0.45	0.31
*Christensenellaceae*	4.45	4.30	4.56	0.17	0.45
*Lachnospiraceae*	4.01	4.09	5.00	0.65	0.62
*Treponema*	2.10^b^	3.85^a^	2.06^b^	0.37	<0.01
*Succiniclasticum*	1.89	1.63	1.52	0.22	0.47
*Oscillospira*	0.88^a^	0.64^b^	0.44^c^	0.03	<0.01
*Mogibacteriaceae*	0.74^a^	0.43^b^	0.27^c^	0.03	<0.01
Others	26.93	15.80	22.85	5.83	0.07

SEM, standard error of the mean.

^a−*c*^Means within a row for doses that do not have a common superscript differ (p <0.05).

**Figure 5:**
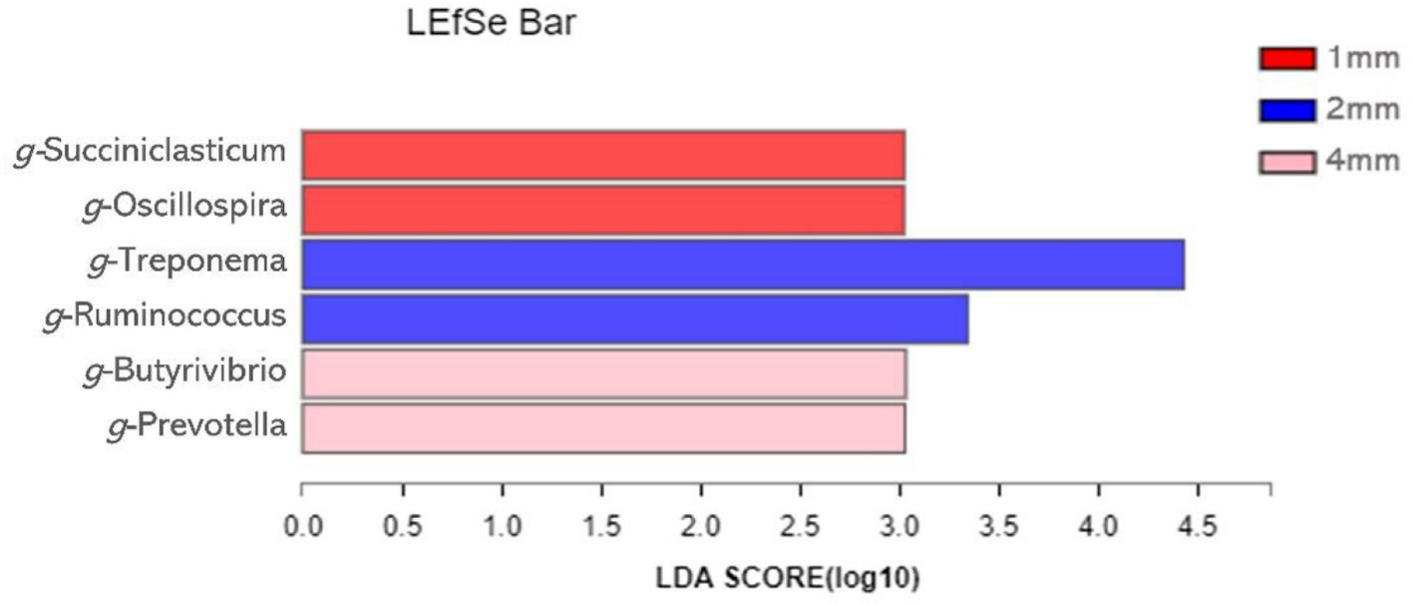
Identification of the most differentially abundant genera in rumen. The plot is generated from Linear Discriminant Analysis Effect Size (LEfSe) analysis with CSS-normalized OTU table and displays taxa with LDA scores above 2 and *p*-values below 0.05.

Linear Discriminant Analysis Effect Size (LEfSe) analysis was used to identify bacteria that were significantly different at the genus level between the three groups (Figure 5). A total of six genera differed significantly between the three groups. The relative abundance of Succiniclasticun and Oscillospira increased in the 1 mm group. The relative abundance of Treponema and Ruminococcus of 2 mm group increased; the relative abundance of Butyrivibrio and Prevotella increased in the 4 mm group.

### 3.5. Microbial correlation analysis

**Figure 6:**
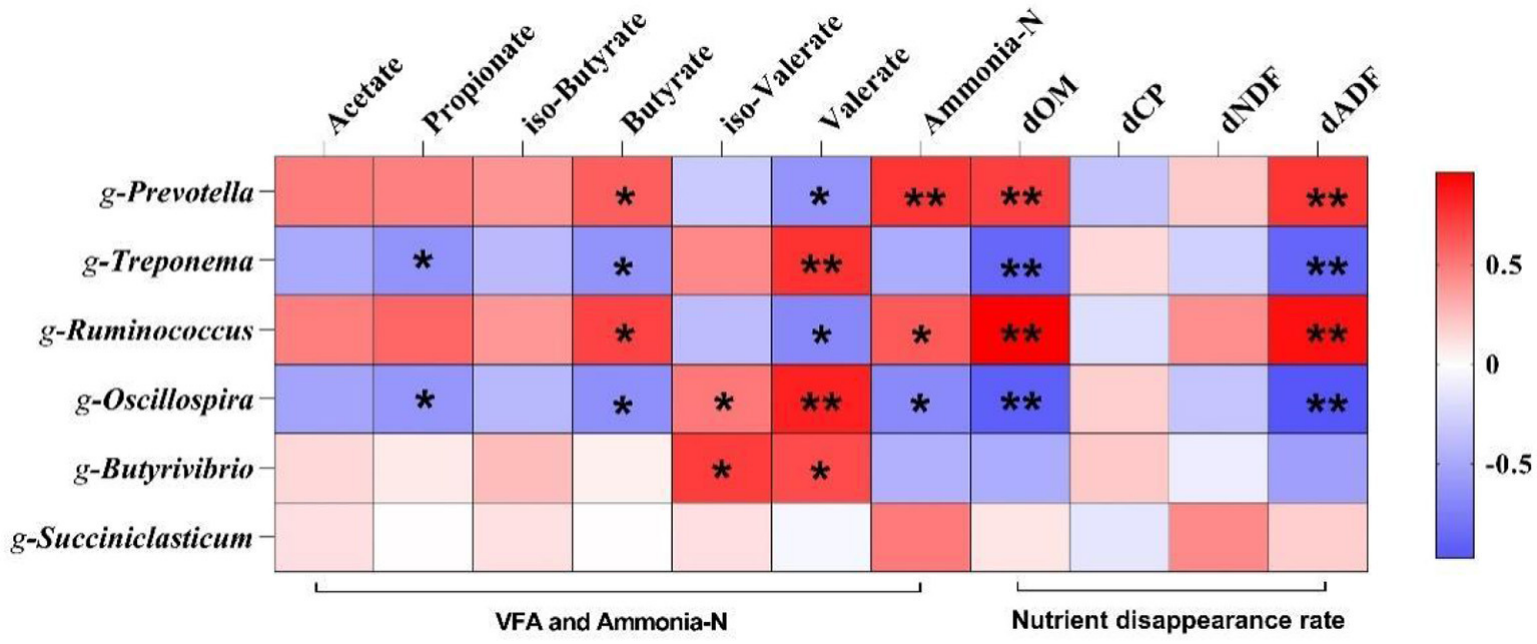
Correlation analysis of volatile fatty acids, ammonia-N and nutrient disappearance rate with and altered microbiota. Significant (*p* < 0.05) or highly significant (*p* < 0.01) statistical differences were represented by * or **. dOM, organic matter disappearance rate; dCP, crude protein disappearance rate; dNDF, neutral detergent fiber disappearance rate; dADF, acid detergent fiber disappearance rate.

To further understand the role of rumen microorganisms in rumen fermentation characteristics and nutrients disappearance rate, six genera with significant differences at the genus level were selected for correlation analysis. As shown in Figure 6, *Prevotella* and *Ruminococcus* was positively correlated with butyrate, ammonia-N, dOM, and dADF (*p* < 0.05) and negatively correlated with valerate (*p* < 0.05); *Treponema* was positively correlated with valerate (*p* < 0.01) and negatively correlated with propionate, butyrate and dOM (*p* < 0.05); *Oscillospira* was positively correlated with valerate (*p* < 0.01) and negatively correlated with propionate, butyrate and ammonia-N (*p* < 0.05); *Butyrivibrio* was positively correlated with iso-valerate and valerate (*p* < 0.05). *Treponema* was positively correlated with valerate (*p* < 0.01) and negatively correlated with propionate, butyrate, dOM, and dADF (*p* < 0.05); *Oscillospira* was positively correlated with valerate (*p* < 0.01) and negatively correlated with propionate, butyrate, ammonia-N, dOM, and dADF (*p* < 0.05); *Butyrivibrio* was positively correlated with iso-valerate and valerate (*p* < 0.05).

## 4. Discussion

Protozoa plays a vital role in the ruminal fermentation of ruminants. Protozoa can phagocytose bacteria, convert dietary fiber and starch into volatile fatty acids, slow down the fermentation rate of carbohydrates in the rumen, and stabilize rumen pH (26). The number of protozoans in this study tended to be stable from day 6 to day 10, which was consistent with the results of Hoover and Knowlton (27). Dietary carbohydrates can produce large amounts of volatile fatty acids under the action of rumen microorganisms. The VFAs are vital energy source for ruminants, providing about 75% of the animal body's energy (28). Previous research results showed that the acetic acid and total VFAs in the large particle size diets group was significantly higher than those in the small particle size diets group in the RUSITEC system (6–8). This was confirmed by the results of the present study, which showed that total VFAs, acetate, propionate, and iso-butyrate contents were higher in the 4 mm group than in the other groups. It may be that a large particle size diets is beneficial to the growth of microorganisms in the rumen, and then promotes the production of volatile fatty acids (28). However, some researchers have found that smaller grain size diets are more conducive to rumen fermentation, which may be closely related to feed and animal species (Table 5).

**Table 5:** Effects of different particle size on rumen fermentation of ruminants *in vitro*.

**Feed type**	**Animals**	**Size**	**Results**	**References**
Wheat straw, Alfalfa hay	Sheep	5 and 0.45 mm	Larger particle size (5 mm) had higher total gas production, acetic acid, total volatile fatty acid, and total nitrogen contents	(29)
Maize silage, Grass silage	Sheep	1 and 4 mm	The relative abundance of microorganisms in the large particle size diet was significantly higher than that in the small particle size diet	(30)
Alfalfa hay	Cows	1 and 3 mm	Fiber content and particle size had no effect on OM and fiber digestion in continuous culture of rumen fluid. However, Larger particle size (4 mm) had higher total gas production, acetic acid, total volatile fatty acid, and total nitrogen contents	(6)
Maize silage, Grass silage	Sheep	1 and 4 mm	Cumulative gas production was recorded during 93 h of incubation and its capacity decreased with increasing proportion of grass silage in the diet, gas production was delayed in 4 mm treatments compared with 1 mm treatments	(7)
Red clover, Gamagrass, and Orchard grass	Holstein heifers	2 and 5 mm	Larger particle size diet is beneficial to the growth of fibrolytic bacteria in the rumen	(31)
Uraria crinita	Lambs	1, 2, 3, 4, 8, and 12 mm	Different particle sizes of uraria crinita can affect the structure and quantity of microbial flora in lamb fermentation broth *in vitro*, thus causing changes in fermentation characteristics. The 2.36 mm particle size provided the best fermentation outcome for 3-month-old lambs	(32)
Hays, Orchard grass, and Alfalfa	Sheep	5.6, 1.18, and 0.3 mm	With the decrease of feed particle size, the contact area between feed and rumen microorganisms will be increased, and the nutrients will be more easily degraded by microorganisms, thus improving the degradation rate of dry matter and crude protein *in vitro*	(33)

Hildebrand et al. (7) used the RUSITEC system to study the effect of 1 and 4 mm particle size diets on rumen fermentation characteristics *in vitro*, and the experimental results showed that the larger particle size diets was more conducive to the fermentation of OM and the decomposition of nutrients. In addition, the results also showed that the content of ammonia-N was higher in the large particle group. Although the results of this experiment also confirmed that larger particle size was beneficial to improving the disappearance rate of OM, but has no effect on the content of ammonia-N. This may be due to the difference research method and diets composition.

Numerous studies have shown that not only nutrients composition of the diets affects the rumen microorganisms, but also the physical structural changes of the diets may alter the interactions between the rumen microorganisms and the animal (34, 35). The different particle sizes of the diets will change the contact area between the diets and rumen microorganisms, which will change the rumen fermentation system (36), then cause the change of rumen microorganisms, and finally affect the rumen fermentation speed and the formation of fermentation products (37). Similar to the results of previous studies (38), this study found that Bacterboidetes and Firmicutes were the dominant phyla. The primary function of *Bacteroidetes* was to degrade carbohydrates and proteins (39, 40). Kaakoush (41) found that Firmicutes carry many genes, producing many digestive enzymes to help animals digest and absorb nutrients. This study found that the relative abundance of Bacteroidetes increased significantly with the increase of rice straw particle sizes, the disappearance rate of OM in 4 mm group was significantly higher than others, which may be related to the increase of relative abundance of *Bacteroidetes*. *Butyrivibrio* was the dominant bacteria in the rumen, which can produce VFAs, CO_2_ and H_2_ by fermenting starch and polysaccharides. *Prevotella* was the main starch degrading bacteria in the rumen, and can decompose and utilize the protein in the diet (39). Fernando et al. (39) have shown that the dominant bacteria in the rumen are not affected by diet, but the results of this study show that the change of diets structure will affect the relative abundance of dominant bacteria genera, such as *Prevotella*, and the specific reasons need to be further explored.

In this study, LEfSe analysis found that the change in rice straw particle size would affect the composition of microorganisms. With the increase in straw particle size, the relative abundance of *Ruminocococus, Butyrivibrio*, and *Prevotella* increase. *Ruminococcus* and *Butyrivibrio* are significant cellulolytic bacteria. In the rumen, they play a crucial role in degrading cellulose and producing VFAs (42, 43). The results of correlation analysis between microorganisms and VFAs and nutrients disappearance rate showed that *Prevotella* was positively correlated with butyrate, ammonia-N, dOM and dADF. Liu et al. (44) found that *Prevotella* is the main protein hydrolysis bacteria in the rumen. The correlation between *Prevotella* and ammonia nitrogen and dOM may be due to the role of *Prevotella* in degrading protein and regulating the rumen environments (45). *Ruminocococus* is the main butyrate-producing bacteria, positively correlated with butyric acid. However, *Ruminocococus* is negatively correlated with valerate, which may be caused by competition between *Ruminocococus* and *Butyrivibrio*, both cellulolytic bacteria. Liu et al. (46) showed that iso-butyric, valerate, and Iso-valerate were beneficial for the growth of fibro catabolic bacteria. The results of this study also show that *Butyrivibrio* was positively correlated with isovalerate and valerate. In summary, compared to the other groups, rice straw particle size of 4 mm may improve the disappearance rate of nutrients and promote the production of VFAs by regulating the structure of rumen microorganisms. However, In the future, more targeted studies are needed to determine the appropriate particle size of the diets, to promote the standardization of RUSITEC system trials and enhance the comparability of different studies.

The results of this study can provide RUSITEC reference for the selection of straw particle size of the diet required by RUSITEC in the fermentation process, and can effectively reduce the experimental error caused by the difference in diet structure. In addition, RUSITEC has more advantages in feed evaluation than ruminants, and optimizing the technical index of RUSITEC is of great significance for the efficient utilization of straw resources in ruminants. However, although this experiment compared straw with three-grain sizes of 1, 2, and 4 mm, it is not clear whether there is a more suitable particle size, and further research is needed.

## 5. Conclusion

The particle sizes of rice straw could significantly affect the nutrients disappearance rate, rumen fermentation, and microbial diversity of the RUSITEC system. This study showed that the nutrient disappearance rate, volatile fatty acids contents, and the relative abundance of beneficial bacteria in the 4 mm group increased compared with other groups. Therefore, compared with other particle sizes, rice straw with a particle size of 4 mm had better fermentation characteristics and nutrients utilization efficiency *in vitro* under the experimental conditions.

## Data availability statement

The datasets presented in this study can be found in online repositories. The names of the repository/repositories and accession number(s) can be found at: https://www.ncbi.nlm.nih.gov/bioproject; PRJNA945584.

## Ethics statement

The animal study was reviewed and approved by Hunan Agricultural University Secretary, Animal Care and Use Committee.

## Author contributions

ZL, HQ, ZW, and WS designed the research. FW, XL, DX, JH, and ZL conducted the research. ZL and HQ analyzed the data. ZL wrote the paper. All authors approved the final manuscript.
